# Antidepressant-Like Effects of* Gyejibokryeong-hwan* in a Mouse Model of Reserpine-Induced Depression

**DOI:** 10.1155/2018/5845491

**Published:** 2018-06-26

**Authors:** Bo-Kyung Park, Yu Ri Kim, Young Hwa Kim, Changsop Yang, Chang-Seob Seo, In Chul Jung, Ik-Soon Jang, Seung-Hyung Kim, Mi Young Lee

**Affiliations:** ^1^Clinical Medicine Division, Korea Institute of Oriental Medicine, Daejeon 34054, Republic of Korea; ^2^Herbal Medicine Research Division, Korea Institute of Oriental Medicine, Daejeon 34054, Republic of Korea; ^3^Department of Oriental Neuropsychiatry, College of Korean Medicine, Daejeon University, Daejeon 34520, Republic of Korea; ^4^Division of Bioconvergence Analysis, Korea Basic Science Institute, Daejeon 34133, Republic of Korea; ^5^Institute of Traditional Medicine and Bioscience, Daejeon University, Daejeon 34520, Republic of Korea

## Abstract

Treatment with the antihypertensive agent reserpine depletes monoamine levels, resulting in depression. In the present study, we evaluated the antidepressant effects of* Gyejibokryeong-hwan* (GBH), a traditional Korean medicine, in a mouse model of reserpine-induced depression. Mice were treated with reserpine (0.5 mg·kg^−1^, i.p.) or phosphate-buffered saline (PBS, i.p., normal) once daily for 10 days. GBH (50, 100, 300, and 500 mg·kg^−1^), PBS (normal, control), fluoxetine (FXT, 20 mg·kg^−1^), or amitriptyline (AMT, 30 mg·kg^−1^) was administered orally 1 h prior to reserpine treatment. Mouse behavior was examined in the forced swim test (FST), tail suspension test (TST), and open-field test (OFT) following completion of the treatment protocol. Administration of GBH reduced immobility time in the FST and TST and significantly increased the total distance traveled in the OFT. Plasma serotonin levels were significantly lower in control mice than in normal mice, although these decreases were significantly attenuated to a similar extent by treatment with GBH, FXT, or AMT. Reserpine-induced increases in plasma corticosterone were also attenuated by GBH treatment. Moreover, GBH attenuated reserpine-induced increases in interleukin- (*IL-) 1β, IL-6,* and tumor necrosis factor- (*TNF-) α* mRNA expression in the hippocampus. In addition, GBH mice exhibited increased levels of brain-derived neurotrophic factor (BDNF) and a higher ratio of phosphorylated cAMP response element-binding protein (p-CREB) to CREB (p-CREB/CREB) in the hippocampus. Our results indicated that GBH can ameliorate depressive-like behaviors, affect the concentration of mood-related hormones, and help to regulate immune/endocrine dysfunction in mice with reserpine-induced depression, likely via activation of the BDNF-CREB pathway. Taken together, these findings indicate that GBH may be effective in treating patients with depression.

## 1. Introduction

Depression is a mood disorder characterized by feelings of unpleasantness, helplessness, sadness, and despair [1]. Such feelings are often accompanied by symptoms such as sleep disturbance, loss of appetite, and decreased concentration, which can substantially impact the patient's quality of life and social functioning. Depression is also a main risk factor for suicide, representing a significant public health concern [2]. World Health Organization (WHO) data have revealed that depression is the fourth greatest contributor to disability-adjusted life years, a parameter representing the number of years lost due to accidents, illness, disability, and premature death. Moreover, researchers have projected that depression will become the second greatest contributor by 2020 [3]. Depression can be divided into several types, including major depressive disorder (MDD), dysthymic disorder, psychotic depression, postpartum depression, seasonal affective disorder (SAD), and bipolar disorder. However, MDD and dysthymic disorder are the most common types of depression [4].

Although various pathological causes of depression have been identified, the monoamine hypothesis is the most well described and accepted [5]. In the 1960s, a neurochemical model of depression was proposed based on reports that monoamine depletion induced by reserpine, a treatment for hypertension, caused adverse effects in patients, leading to depression [6]. These data suggest that depression is associated with monoaminergic dysfunction in the central nervous system. Many antidepressant therapies targeting monoamines, such as tricyclic antidepressants (TCAs), selective serotonin reuptake inhibitors (SSRIs), noradrenaline reuptake inhibitors (NRIs), serotonin and noradrenaline reuptake inhibitors (SNRIs), and monoamine oxidase inhibitors (MAOIs), have been developed based on this theory [7]. However, these antidepressants are often associated with anticholinergic or neurological side effects such as dizziness, sedation, sexual dysfunction, insomnia, and anxiety. Therefore, much research has focused on identifying natural and alternative therapies for depression with fewer side effects [8].

*Gyejibokryeong-hwan* (GBH) is a traditional Korean medicine described in the* Donguibogam*, a traditional textbook of Korean medicine [9]. GBH is comprised of herbs including* Ramulus Cinnamomi cassia*,* Scierotium Poriae cocos*,* Radix Albus paeoniae Lactiflorae*,* Cortex Radicis moutan*, and* Semen Pruni persicae* [10]. GBH has been used extensively to treat blood stasis and climacteric syndrome throughout Asia [11, 12] and has been approved by both the Korean Ministry of Food and Drug Safety (MFDS) and the US Food and Drug Administration (FDA). Additional studies have investigated the safety and efficacy of GBH in various populations [13, 14].

*Ramulus Cinnamomi cassia *exhibits anti-inflammatory effects in murine BV2 microglia cells [15], while* Cortex Radicis moutan* reduces oxidative stress in rat pheochromocytoma PC12 cells [16]. Moreover, amygdalin, a component of* Semen Pruni Persicae,* induces neurotrophic effects* via* the activation of the ERK1/2 pathway in PC12 cells [17]. However, to the best of our knowledge, no studies have investigated the therapeutic potential of GBH for depression. In the present study, we aimed to assess the antidepressant effect of GBH in animal models of depression, in which monoamine depletion was induced using reserpine.

## 2. Materials and Methods

### 2.1. Preparation of GBH

GBH was purchased from Hanpoong Pharm and Foods Co., Ltd. (Jeonju, Korea). A voucher specimen of the herb sample was deposited in the Herbarium of Hanpoong Pharm and Foods Co., Ltd. The component herbs of GBH (ratio of five herbs = 1:1:1:1:1; total weight = 1.7 kg) were extracted in water after boiling for 3 h. The GBH extract was then filtered and vacuum-concentrated, resulting in a yield of 29.53%. The extract was stored at −80°C and dissolved in phosphate-buffered saline (PBS) before use.

### 2.2. High-Performance Liquid Chromatography (HPLC) Analysis of GBH

The GBH extract was analyzed using a Shimadzu Prominence LC–20A system (Kyoto, Japan), which consisted of a solvent delivery unit, an online degasser, a column oven, a sample autoinjector, and a photodiode array (PDA) detector. The data were acquired and processed using LCsolution software (Version 1.24, SP1, Kyoto, Japan). Amygdalin (PubChem CID: 656516, purity 99.0%), gallic acid (PubChem CID: 370, purity 99.0%), and coumarin (PubChem CID: 323, purity 99.0%) were purchased from Merck KGaA (Darmstadt, Germany). Albiflorin (PubChem CID: 51346141, purity 99.8%), paeoniflorin (PubChem CID: 442534, purity 98.8%), cinnamic acid (PubChem CID: 444539, purity 99.0%), and paeonol (PubChem CID: 11092, purity 99.9%) were obtained from Wako (Osaka, Japan). The eight marker components were separated on a Waters SunFire C_18_ column (250 × 4.6 mm, 5 *μ*m, Milford, MA, USA) and maintained at 40°C. The mobile phases consisted of water (A) and acetonitrile (J. T. Baker, Phillipsburg, NJ, USA) (B), both containing 1.0% (v/v) acetic acid (Merck KGaA, Darmstadt, Germany). The gradient elution of the mobile phase was as follows: 10–60% B for 0–30 min, 60–100% B for 30–40 min, 100% B for 40–45 min, and 100–10% B for 45–50 min. The flow-rate and injection volume were 1.0 ml/min and 10 *μ*l, respectively.

### 2.3. Animal Experiments

Seven-week-old male C57BL/6 mice were purchased from Daehan Biolink Co. (Chungbuk, Korea). Animal experiments were performed in accordance with the National Institutes of Health (NIH) Guide for the Care and Use of Laboratory Animals and approved by the Korean Institute of Oriental Medicine Institutional Animal Care and Use Committee (written approval number: 17-081). The mice were acclimated for 1 week, following which depression was induced by administering reserpine (0.5 mg·kg^−1^ in PBS; i.p.; Sigma-Aldrich, St Louis, MO, USA) once per day for 10 days. Normal mice were injected with PBS alone. The reserpine-treated mice were divided into seven groups (n = 6 per group) and orally treated with PBS (Control), GBH (50, 100, 300, and 500 mg·kg^−1^), the SSRI fluoxetine (FXT; 20 mg·kg^−1^; Sigma-Aldrich), or the TCA amitriptyline (AMT; 30 mg·kg^−1^; Sigma-Aldrich). Normal mice were treated with oral doses of PBS. The experimental schematic, including reserpine induction and administration schedules, is presented in Figure 2(a).

### 2.4. Alterations in Body Weight and Food Intake

Body weight was measured on days 1, 5, and 10. Food intake was estimated as the difference between the amount of food remaining in the feeder on day 5 or 10 and the amount of food provided on day 1 [18].

### 2.5. Behavioral Tests

The forced swim test (FST) is used to evaluate learned helplessness in rodents and has often been used to examine the effect of antidepressants in animal models of depression. Mice were allowed to swim for 15 min (session 1) on the day prior to the FST [19]. On test day, individual mice were introduced into a cylinder (height: 45 cm; diameter: 20 cm) containing tap water (25 ± 2°C; depth: 25 cm) from which they could not escape and had to swim to stay afloat. Total immobility time was measured during the last 4 min of a 6 min trial using video tracking software (SMART 3.0; Panlab S.I., Barcelona, Spain).

The tail suspension test (TST) is a useful behavioral tool for examining the effects of antidepressant drugs [20]. Mice were acoustically and visually isolated, following which they were suspended 50 cm above the floor using adhesive tape placed approximately 1 cm from the tip of the tail. Immobility time was recorded during the last 4 min of a 6 min session using the same video tracking software used in the FST.

The open-field test (OFT) can be used to measure anxiety and locomotor behavior in rodents [21]. In the present study, the open-field arena (30 × 30 cm) was constructed from acrylic sheets, and mice were placed individually in the center of the field. Their behavior was recorded for 10 min. Recordings were analyzed using video tracking software (EthoVision XT 9.0, Noldus Information Technology, Wageningen, Netherlands), as described by Deussing [22].

Mice underwent behavioral testing in the following order, with a 6 h interval between each experiment: OFT, TST, FST.

### 2.6. Enzyme-Linked Immunosorbent Assay (ELISA)

Mouse blood was collected into heparin coated tubes under anesthesia with tiletamine/zolazepam (25 mg·kg^−1^, Zoletil 50; Virbac, Cedex, France). For plasma collection, the samples were centrifuged at 3,000 rpm for 10 min at 4°C, following which the supernatant was carefully transferred to a new tube. Plasma samples were stored at −80°C before use. The concentrations of plasma serotonin (Abcam, Cambridge, UK) and corticosterone (Cayman chemical company, Ann Arbor, MI, USA) were determined using ELISA kits, in accordance with the manufacturer's protocols.

### 2.7. Real-Time Polymerase Chain Reaction (PCR)

Total RNA was isolated using Trizol reagent (Invitrogen), while cDNA synthesis was performed using the PrimeScript™ RT reagent kit (TaKaRa, Shiga, Japan).* Interleukin- (IL-) 1β, IL-6, tumor necrosis factor- (TNF-) α,* and* glyceraldehyde-3 phosphate dehydrogenase* (*GAPDH*) mRNA was quantified using a QuantStudio™ 6 Flex real-time polymerase chain reaction (real-time PCR) system (Applied Biosystems, CA, USA) with Power SYBR® Green PCR Master Mix (Applied Biosystems) [23]. The sequences of the real-time PCR primers are presented in Table 1.

### 2.8. Western Blotting

The hippocampus was homogenized in 300 *μ*l lysis buffer (Pro-Prep™; Intron Biotechnology, Korea) containing 1 mM PMSF and 1 *μ*g·ml^−1^ of protease inhibitor mix. Equal amounts (20 *μ*g) of protein were separated using 10% sodium dodecyl sulfate-polyacrylamide gel electrophoresis (SDS-PAGE) and transferred to polyvinylidene fluoride (PVDF) membranes (Amersham Biosciences, Piscataway, NJ, USA). The membranes were then blocked in 5% skim milk in Tris-buffered saline with 0.1% TWEEN® 20 (TBS/T) for 1 h. The membranes were probed overnight with antibodies for brain-derived neurotrophic factor (BDNF; Abcam, Cambridge, UK), phosphorylated cAMP response element-binding protein (p-CREB), and CREB (Cell Signaling Technology, Inc., Danvers, MA, USA) at 4°C. Next, blots were incubated in horseradish peroxidase- (HRP-) conjugated secondary antibody for 1 h at 25°C, and HRP was detected using a chemiluminescent detection reagent (Amersham Biosciences). *β*-Actin (Sigma-Aldrich) was used as a loading control [24].

### 2.9. Immunofluorescence

The brain was frozen at –20°C, and sections were cut to a thickness of 20 *μ*m using a Cryostat Microtome (CM 3050 S, Leica Microsystems, Wetzlar, Germany). We performed double immunofluorescence staining by incubating tissue sections with antibodies for BDNF (Novus Biologicals, Inc., Littleton, CO), p-CREB, and CREB (Cell Signaling Technology, Inc.) overnight at 4°C. Subsequently, FITC-conjugated secondary antibody was added for 2 h, and nuclear staining was performed using DAPI. Sections were observed using an Eclipse T*i*-E inverted fluorescent microscope (Nikon Instruments Inc., Mississauga, Canada).

### 2.10. Statistical Analysis

All data are expressed as the mean ± standard deviation (SD). One-way analysis of variance (ANOVA) was performed using GraphPad Prism version 7 (GraphPad Software Inc., San Diego, CA, USA) to assess between-group differences. Comparisons among multiple groups were performed using one-way ANOVAs, followed by* post hoc* Tukey tests. The level of statistical significance was set at p < 0.05.

## 3. Results

### 3.1. HPLC Analysis of GBH

**Figure 1:**
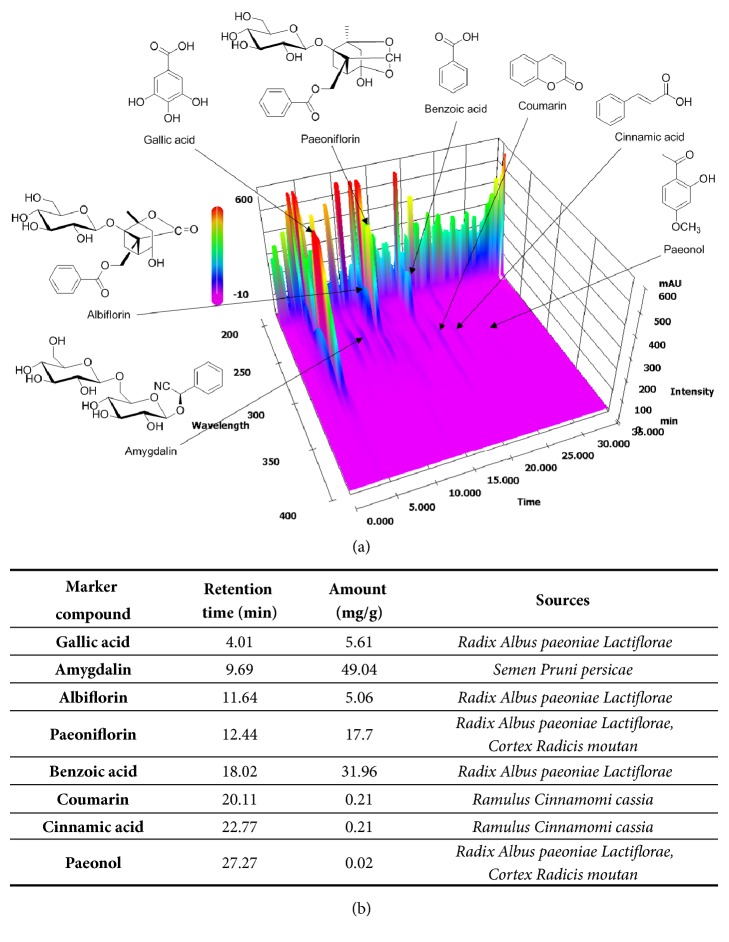
Three-dimensional chromatogram of* Gyejibokryeong-hwan* (GBH) sample based on HPLC–PDA analysis. HLPC: high-performance liquid chromatography; PDA: photodiode array.

The composition of GBH was verified using HPLC. This analysis provided chemical information regarding GBH, guaranteeing the reproducibility of our experiments using other batches of GBH. Analyses were performed in accordance with chemical standards described by Kim et al. [14]. Quantitative and qualitative analyses of the eight marker compounds (gallic acid, amygdalin, albiflorin, paeoniflorin, benzoic acid, coumarin, cinnamic acid, and paeonol) in GBH were conducted using the optimized HPLC–photodiode array (PDA) method. Each GBH component was identified by comparing the retention time and UV spectra with the respective reference standards. The retention times and amounts of the eight marker compounds in GBH are shown in Figure 1.

### 3.2. Effect of GBH on Body Weight and Food Intake

**Figure 2:**
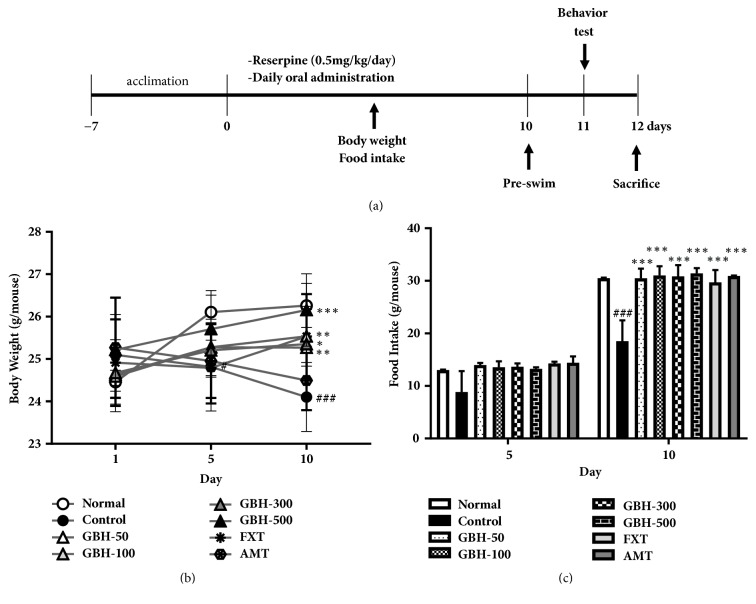
Effect of* Gyejibokryeong-hwan* (GBH) on development of reserpine-induced depression in mice. Mice underwent oral treatment with vehicle (PBS), GBH (50, 100, 300, or 500 mg·kg^−1^), fluoxetine (FXT, 20 mg·kg^−1^), or amitriptyline (AMT, 30 mg·kg^−1^) once per day for 10 days. (a) Schematic showing reserpine treatment, oral administration schedule, and behavioral testing timeline. (b) Body weight and (c) food intake were measured on days 1, 5, and 10. Data are presented as the mean ± standard deviation (SD) (n = 6, one-way ANOVA; ^#^p < 0.05, ^###^p < 0.001 versus normal; *∗*p < 0.05, *∗∗*p < 0.01, *∗∗∗*p < 0.001 versus control).

No significant differences in mean body weight were observed among the groups on day 1. After 5 and 10 days of reserpine injection, body weight was significantly lower in control mice than in normal mice; however, oral GBH administration ameliorated this effect after 10 days of treatment (GBH 100: 25.54 ± 0.71 g, p < 0.01; GBH 300: 25.35 ± 0.82 g, p < 0.05; and GBH 500: 26.16 ± 0.85 g, p < 0.001; Figure 2(b)). Ten days of FXT administration (20 mg·kg^−1^) significantly increased body weight (FXT: 25.53 ± 1.0 g, p < 0.01; Figure 2(b)); however, there was no difference in body weight following AMT administration (30 mg·kg^−1^), relative to that observed in control mice.

Food intake was significantly lower in control mice than in normal mice on day 10 (18.5 ± 3.97 g, p < 0.001); however, administration of GBH, FXT, or AMT significantly ameliorated this effect (GBH 50: 30.49 ± 1.83 g; GBH 100: 31.02 ± 1.76 g; GBH 300: 30.83 ± 2.14 g; GBH 500: 31.4 ± 1.01 g; FXT: 29.71 ± 2.33 g; AMT: 30.9 ± 0.13 g; all p < 0.001; Figure 2(c)). These results suggest that GBH counteracted decreases in body weight and food intake in mice with reserpine-induced depression.

### 3.3. Effect of GBH on Depressive-Like Behavior

**Figure 3:**
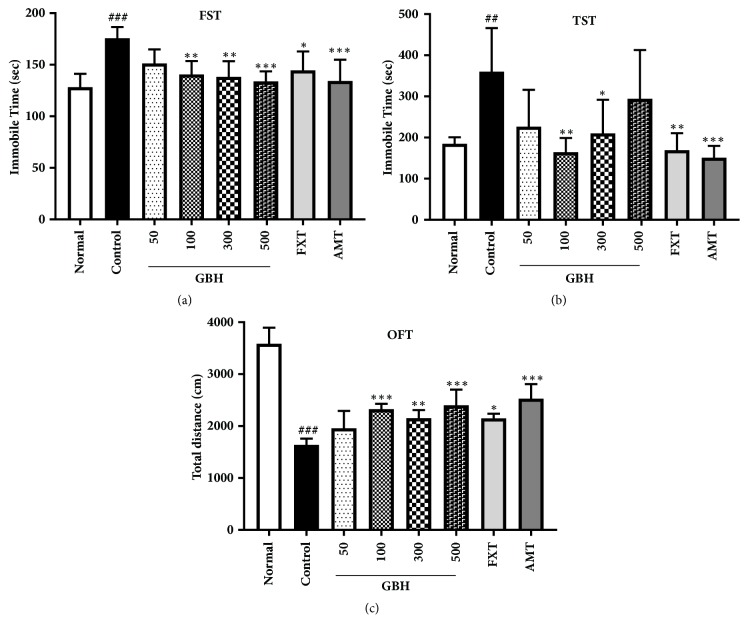
Effect of* Gyejibokryeong-hwan* (GBH) on reserpine-induced depressive-like behaviors in mice. (a) Immobility time in the forced swim test (FST) and (b) tail suspension test (TST); (c) total distance traveled in the open-field test (OFT). All behavioral tests were conducted on day 11. Data are presented as the mean ± standard deviation (SD) (n = 6, one-way ANOVA; ^##^p < 0.01, ^###^p < 0.001 versus normal; *∗*p < 0.05, *∗∗*p < 0.01, *∗∗∗*p < 0.001 versus control).

We measured immobilization time in the FST (F[7, 40] = 6.09, p < 0.001; Figure 3(a)) 1 day after the completion of treatment (day 11; Figure 2(a)). As expected, immobilization time was significantly higher in control mice than in normal mice (control: 175.68 ± 10.87 s, p < 0.001). Moreover, immobilization time was significantly lower in GBH mice than in control mice (GBH 100: 140.57 ± 13.06 s, p < 0.01; GBH 300: 138.19 ± 15.3 s, p < 0.01; GBH 500: 133.88 ± 9.72 s, p < 0.001; Figure 3(a)). Furthermore, treatment with FXT and AMT significantly decreased immobilization time (FXT: 144.55 ± 18.36 s, p < 0.05; AMT: 134.26 ± 20.63 s, p < 0.001).

Mice treated with GBH exhibited significant decreases in immobilization time during the TST (F[7,40] = 5.754, p < 0.001; Figure 3(b)) (GBH 100: 164.53 ± 34.28 s, p < 0.01; GBH 300: 210.4 ± 81.54 s, p < 0.05) when compared with control mice (control: 360.0 ± 106.2 s, p < 0.01 versus normal; Figure 3(b)).

Distance traveled in the OFT (F[7,40] = 36.41, p < 0.001; Figure 3(c)) was significantly greater in mice treated with GBH (GBH 100: 2325.64 ± 102.26 cm, p < 0.001; GBH 300: 2150.78 ± 158.16 cm, p < 0.01; GBH 500: 2401.32 ± 301.27 cm, p < 0.001) than in control mice (control: 1637.27 ± 121.65 cm, p < 0.001 versus normal). In addition, FXT and AMT treatment significantly increased the distance traveled (FXT: 2147.08 ± 89.7 cm, p < 0.05; AMT: 2528.21 ± 280.07 cm, p < 0.001; Figure 3(c)). These results suggest that GBH can ameliorate depressive-like behaviors in mice with reserpine-induced depression.

### 3.4. Effect of GBH on Mood-Related Hormones in Reserpine-Treated Mice

**Figure 4:**
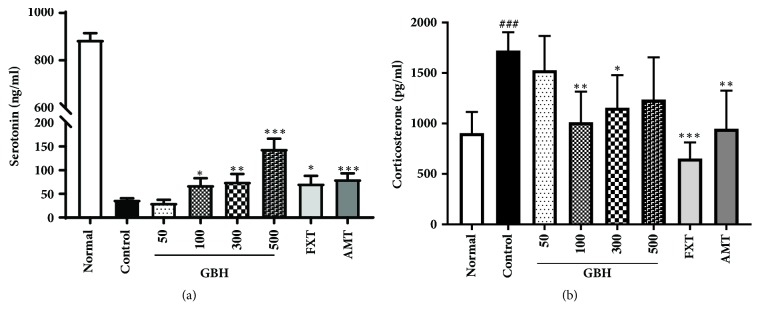
Effect of* Gyejibokryeong-hwan* (GBH) on the concentration of plasma serotonin and corticosterone in mice with reserpine-induced depression. The concentrations of plasma (a) serotonin and (b) corticosterone were determined via ELISA. Data are presented as the mean ± standard deviation (SD) (n = 6, one-way ANOVA; ^###^p < 0.001 versus normal; *∗*p < 0.05, *∗∗*p < 0.01, *∗∗∗*p < 0.001 versus control).

The concentration of plasma serotonin, a key regulator of emotions and mood disorders [25], was significantly decreased in control mice following reserpine administration (control: 37.91 ± 2.73 ng/ml, p < 0.001 versus normal). However, treatment with GBH, FXT, and AMT attenuated these decreases (GBH 100: 68.84 ± 14.2 ng/ml, p < 0.05; GBH 300: 75.82 ± 16.15 ng/ml, p < 0.01; GBH 500: 145.52 ± 20.96 ng/ml, p < 0.001; FXT: 72.3 ± 15.65 ng/ml, p < 0.05; AMT: 81.19 ± 11.97 ng/ml, p < 0.001; Figure 4(a)). Moreover, treatment with GBH, FXT, and AMT significantly attenuated the reserpine-induced increase in serum levels of the stress hormone corticosterone (GBH 100: 1013.04 ± 303.21 pg/ml, p < 0.01; GBH 300: 1155.06 ± 324.15 pg/ml, p < 0.05; FXT: 652.59 ± 160.33 pg/ml, p < 0.001; AMT: 947.41 ± 377.8 pg/ml, p < 0.01) [26], relative to levels observed in control mice (control: 1722.91 ± 181.69 pg/ml, p < 0.001 versus normal; Figure 4(b)). These data suggest that GBH can affect the concentration of mood-related hormones in mice with reserpine-induced depression. Dopamine and norepinephrine were not detected in the plasma (data not shown).

### 3.5. Effect of GBH on Proinflammatory Cytokines in Reserpine-Induced Depression

**Figure 5:**
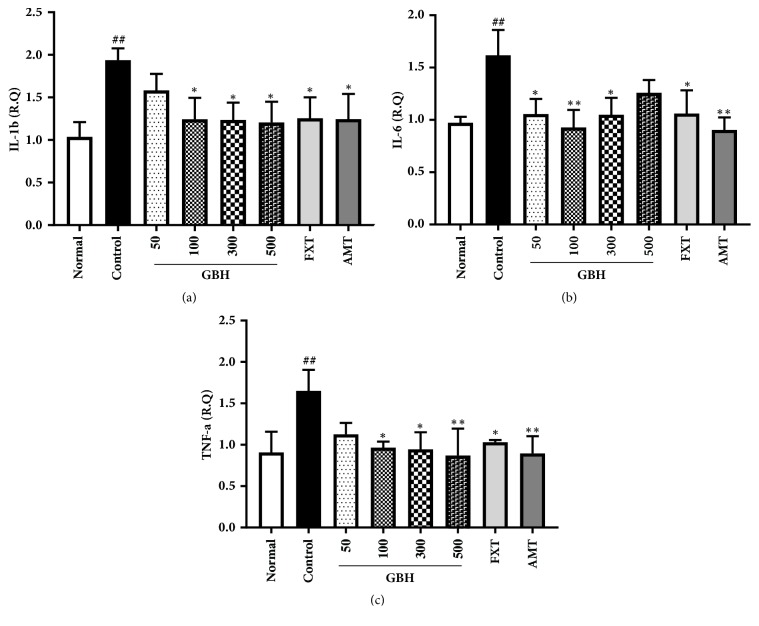
Effect of* Gyejibokryeong-hwan* (GBH) on* IL-1β, IL-6, *and* TNF-α* mRNA expression in mice with reserpine-induced depression. The expression of (a)* IL-1β,* (b)* IL-6, *and (c)* TNF-α* mRNA was determined via quantitative real-time PCR. Data represent mean ± standard deviation (SD) (n = 6, one-way ANOVA; ^##^p < 0.01 versus normal; *∗*p < 0.05, *∗∗*p < 0.01 versus control). IL-1*β*: interleukin 1 beta; IL-6: interleukin 6; TNF-*α*: tumor necrosis factor alpha; PCR: polymerase chain reaction.

Chronic stress and the release of proinflammatory cytokines such as IL-1*β*, IL-6, and TNF-*α* lead to chronic neuroinflammation, which contributes to depression [27, 28].* IL-1β, IL-6, *and* TNF-α* mRNA expression was significantly increased in the hippocampus of control mice, although this effect was ameliorated by treatment with GBH, FXT, and AMT (Figure 5). These data indicate that GBH may help to regulate immune and endocrine dysfunction associated with depression.

### 3.6. Effect of GBH on BDNF and p-CREB Expression in the Brain

**Figure 6:**
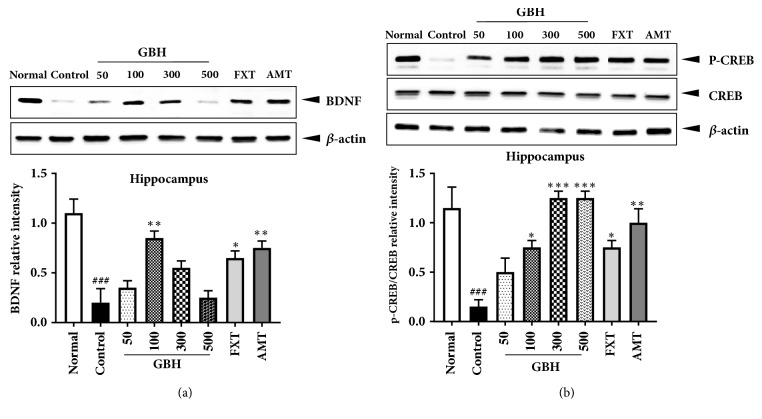
Effect of* Gyejibokryeong-hwan* (GBH) on hippocampal levels of BDNF and p-CREB/CREB, as measured via Western blotting, in mice with reserpine-induced depression. Isolated hippocampal lysates were analyzed via Western blotting using (a) BDNF and (b) p-CREB/CREB antibodies. *β*-Actin was used as the loading control. Data are representative of three independent experiments and are presented as the mean ± standard deviation (SD) (one-way ANOVA; ^###^p < 0.001 versus normal; *∗*p < 0.05, *∗∗*p < 0.01, *∗∗∗*p < 0.001 versus control). BDNF: brain-derived neurotrophic factor; CREB: cAMP response element-binding protein; p-CREB: phosphorylated CREB.

In order to determine the molecular mechanisms underlying the antidepressant effects of GBH, we examined BDNF and p-CREB expression in the brain via Western blot analysis. Hippocampal BDNF levels were significantly lower in control mice than in normal mice, indicative of neuronal dysfunction in the brain [29]; however, mice in the GBH 100 group exhibited significantly increased levels of BDNF (Figure 6(a)). The BDNF-CREB pathway is associated with MDD [30]. We observed a significant reduction in hippocampal p-CREB in control mice, which was prevented in a dose-dependent manner in mice treated with GBH. Moreover, FXT and AMT treatment significantly increased p-CREB expression (Figure 6(b)). These results suggest that GBH can affect hippocampal neuronal activity in mice with reserpine-induced depression.

**Figure 7:**
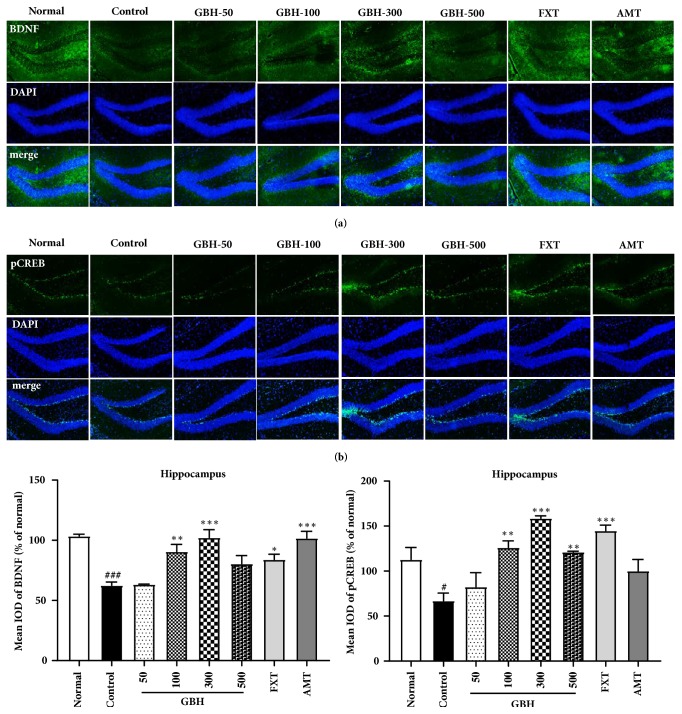
Effect of* Gyejibokryeong-hwan* (GBH) on hippocampal BDNF and p-CREB, as measured using immunofluorescence, in mice with reserpine-induced depression. Frozen hippocampal sections were analyzed based on immunofluorescence using (a) BDNF (green) and (b) p-CREB (green) antibodies. DAPI (blue) was used to visualize nuclei. Data are representative of three independent experiments and are presented as the mean ± standard deviation (SD) (one-way ANOVA; ^#^p < 0.05, ^###^p < 0.001 versus normal; *∗*p < 0.05, *∗∗*p < 0.01, *∗∗∗*p < 0.001 versus control). BDNF: brain-derived neurotrophic factor; p-CREB: phosphorylated cAMP response element-binding protein.

To confirm the effect of GBH treatment in our model of reserpine-induced depression, we examined BDNF and p-CREB expression in the hippocampus via immunofluorescence analysis. BDNF levels were significantly decreased in the dentate gyrus of control mice, although this decrease was attenuated by treatment with GBH, FXT, and AMT (Figure 7(a)). Similar results were observed when we examined changes in p-CREB expression (Figure 7(b)). These results suggest that GBH recovers reserpine-induced depressive-like behaviors via activation of the BDNF-CREB pathway.

## 4. Discussion

In the present study, we examined the antidepressant effect of GBH in mice with reserpine-induced depression. Our results indicated that GBH can ameliorate depressive-like behaviors, affect the concentration of mood-related hormones, and help to regulate immune/endocrine dysfunction in mice with reserpine-induced depression, likely via activation of the BDNF-CREB pathway.

As there are various causes of depression, the pathophysiology of the disorder remains to be fully elucidated. Several hypotheses have been proposed regarding the basis of depression, including hypothalamic-pituitary-adrenal (HPA) axis hyperactivity [31]; disturbances in monoamine, glutamate, and gamma-aminobutyric acid (GABA) transmission; neurotrophic factor dysfunction; neuroinflammation [32]; and glial pathology [33, 34].

Monoamines are transported into presynaptic vesicles via the vesicular monoamine transporter (VMAT), which is blocked by reserpine [35]. Mice with reserpine-induced depression due to monoamine depletion exhibit anxiety- and depressive-like behaviors, such as increased immobility time and decreased locomotor activity in behavioral tests, relative to findings observed in control mice [36]. In addition, previous studies have reported that mice treated with reserpine exhibit increased levels of plasma corticosterone [37] and proinflammatory cytokines in the brain [38].

Before initiating our study, we optimized our mouse model of reserpine-induced depression. Our findings indicated that higher concentrations of reserpine administered for 3 consecutive days were lethal in some mice prior to the 10-day GBH treatment period. Furthermore, some mice recovered from reserpine-induced depression 10 days after drug treatment, while treatment at lower doses for 3 days was not sufficient for inducing depression. In the present study, we modified the animal systems used in previous studies [36] by reducing the concentration of reserpine to 0.5 mg·kg^−1^. In addition, the reserpine treatment time was extended to 10 days, and GBH was administered simultaneously.

Our results indicated that GBH successfully ameliorated depressive-like behaviors in reserpine-treated mice, as indicated by significant reductions in immobility time in the FST and TST. Furthermore, our findings demonstrated that such decreases were not due to increases in spontaneous locomotor activity (Supplementary Material 1). However, GBH did increase the distance traveled in the OFT, indicating that GBH can suppress anxiety-related behavior in mice with reserpine-induced depression.

Reserpine blocks amine storage processes, which leads to increased hippocampal excitability and blood corticoid levels. Previous studies have demonstrated that reserpine alters the secretion of 5-hydroxytryptamine (5-HT) in the brain [37]. In the present study, we observed that GBH treatment significantly increased plasma serotonin levels while significantly decreasing corticosterone levels in reserpine-treated mice, relative to levels observed in control mice. Although plasma serotonin levels may represent release from both the central nervous system and platelets, GBH is known to suppress blood aggregation—a process that activates serotonin release [39, 40]. Thus, it is unlikely that platelets represented the major source of serotonin release in our study. Although the recovery of plasma serotonin levels was significantly greater in the GBH-treated group than in the control group, the relative serotonin level in the GBH-treated group was still significantly lower than that in the naive group.

The balance of extracellular catecholamines is a key modulator of inflammatory mediator production [38, 41]; therefore, we assessed the expression of proinflammatory cytokine mRNA in the hippocampus. Our findings indicated that GBH treatment attenuated reserpine-induced increases in the expression of* IL-1β, IL-6,* and* TNF-α* mRNA in control mice.

An overproduction of proinflammatory cytokines can impair neuronal structure and function, leading to deficits in neuroplasticity [42]. BDNF and CREB are involved in neuronal differentiation, survival, and synaptic plasticity—which are associated with learning and memory—and in many nervous system disorders, including depression [30]. Western blot and immunofluorescence analyses revealed that BDNF and p-CREB expression in the hippocampus was greater in GBH-treated mice than in control mice. Because treatment with GBH at 300 and 500 mg·kg^−1^ resulted in gradual decreases in BDNF levels, our findings suggest that the optimal concentration of GBH for improving BDNF expression is 100 mg·kg^−1^. Such results may have been associated with the pharmacokinetic properties of GBH, which is comprised of extracts from five types of plants. Indeed, such properties are often unpredictable in multicompound herbal medicines. For example, an unidentified chemical compound in GBH may exert adverse effects on BDNF levels beyond a certain threshold, decreasing the efficacy of GBH treatment. Our data suggest that GBH not only exerts regulatory effects on neuroinflammation but also influences BDNF expression in the brain.

Recently developed antidepressants include modulators of neuroinflammation, oxidative stress, the hypothalamic-pituitary-adrenal (HPA) axis, glutamate, opioids, the cholinergic system, and neuropeptides (e.g., substance P, neuropeptide Y, and galanin). In addition, many studies have sought to treat depression without side effects [43, 44]. Natural medicines with anti-inflammatory and antioxidant properties are attractive targets because they have been effective in treating neurodegenerative and autoimmune diseases, as well as cancer. Moreover, they are associated with significantly fewer side effects (e.g., St. John's wort [*Hypericum perforatum*]) [45, 46]. Toxicity studies have demonstrated that GBH is safe at dosages up to 500 mg·kg·day^−2^, regardless of gender. Furthermore, standardization results have been reported in accordance with Ministry of Food and Drug Safety (MFDS) guidelines [47].

## 5. Conclusions

Taken together, our results indicate that GBH treatment can induce an antidepressant-like effect in mice with reserpine-induced depression. GBH is composed of several herbs; therefore, our results suggest that a multitarget approach can be effective in treating depression with fewer side effects. Further studies are required to determine the active compound(s) in GBH, as well as the molecular mechanisms underlying its regulatory effects on neuroinflammation and HPA axis hyperactivity.

## Figures and Tables

**Table 1:** The sequences of the real-time PCR primers

Gene	Sequence
mouse *IL-1β*	forward, 5′- GCTGAAAGCTCTCCACCTCA -3′ reverse, 5′- AGGCCACAGGTATTTTGTCG -3′

mouse *IL-6*	forward, 5′- GAGGATACCACTCCCAACAGACC -3′ reverse, 5′- AAGTGCATCATCGTTGTTCATACA -3′

mouse *TNF-α*	forward, 5′- AGACCCTCACACTCAGATCATCTTC -3′ reverse, 5′- CCACTTGGTGGTTTGCTACGA -3′

mouse *GAPDH*	forward, 5′- AAGGTGGTGAAGCAGGCAT -3′ reverse, 5′- GGTCCAGGGTTTCTTACTCCT -3′

## Data Availability

The datasets used and/or analyzed in the current study are available from the corresponding author upon reasonable request. The role of the funding body in the design of the study and collection, analysis, and interpretation of data and in writing the article should be declared in this request.

## Abbreviations

MDD: Major depressive disorder
SAD: Seasonal affective disorder
TCAs: Tricyclic antidepressants
SSRIs: Selective serotonin reuptake inhibitors
NRIs: Noradrenaline reuptake inhibitors
SNRIs: Serotonin and noradrenaline reuptake inhibitors
MAOIs: Monoamine oxidase inhibitors
GBH: Gyejibokryeong-hwan
BDNF: Brain-derived neurotrophic factor
PBS: Phosphate-buffered saline
FST: Forced swimming test
TST: Tail suspension test
OFT: Open-field test
p-CREB: Phosphorylated cAMP response element-binding protein
HRP: Horseradish peroxidase
IL: Interleukin
TNF: Tumor necrosis factor
HPA: Hypothalamic-pituitary-adrenal
WHO: World Health Organization
MFDS: Ministry of Food and Drug Safety
FDA: Food and Drug Administration
IP: Intraperitoneal
GABA: Gamma-aminobutyric acid
5-HT: 5-hydroxytryptamine
SD: Standard deviation
FXT: Fluoxetine
AMT: Amitriptyline
VMAT: Vesicular monoamine transporter.

## Supplementary Materials

Supplementary Materials We performed behavior test and ELISA in which normal mice were treated with GBH at dosages of 100, 300, and 500 mg/kg for 10 days. The GBH did not show a significant effect in these experiments. Supplementary Material 1: effect of* Gyejibokryeong-hwan* (GBH) on depressive-like behaviors and the concentration of plasma serotonin and corticosterone in mice. (a) Immobility time in the forced swim test (FST) and (b) tail suspension test (TST); (c) total distance traveled in the open-field test (OFT). All behavioral tests were conducted on day 11. The concentration of plasma (d) serotonin and (e) corticosterone was determined by ELISA. Data represent mean ± SD (n = 6, one-way ANOVA).
