# Supplementary material for: Antidepressant-Like Effects of Gyejibokryeong-hwan in a Mouse Model of Reserpine-Induced Depression
Source: Biomed Res Int. 2018 Jun 26;2018:5845491. doi: 10.1155/2018/5845491 (PMC6038693; doi:10.1155/2018/5845491)
Supplement: Supplementary Materials — We performed behavior test and ELISA in which normal mice were treated with GBH at dosages of 100, 300, and 500 mg/kg for 10 days. The GBH did not show a significant effect in these experiments. Supplementary Material 1: effect of Gyejibokryeong-hwan (GBH) on depressive-like behaviors and the concentration of plasma serotonin and corticosterone in mice. (a) Immobility time in the forced swim test (FST) and (b) tail suspension test (TST); (c) total distance traveled in the open-field test (OFT). All behavioral tests were conducted on day 11. The concentration of plasma (d) serotonin and (e) corticosterone was determined by ELISA. Data represent mean ± SD (n = 6, one-way ANOVA). [file 5845491.f1.pdf]

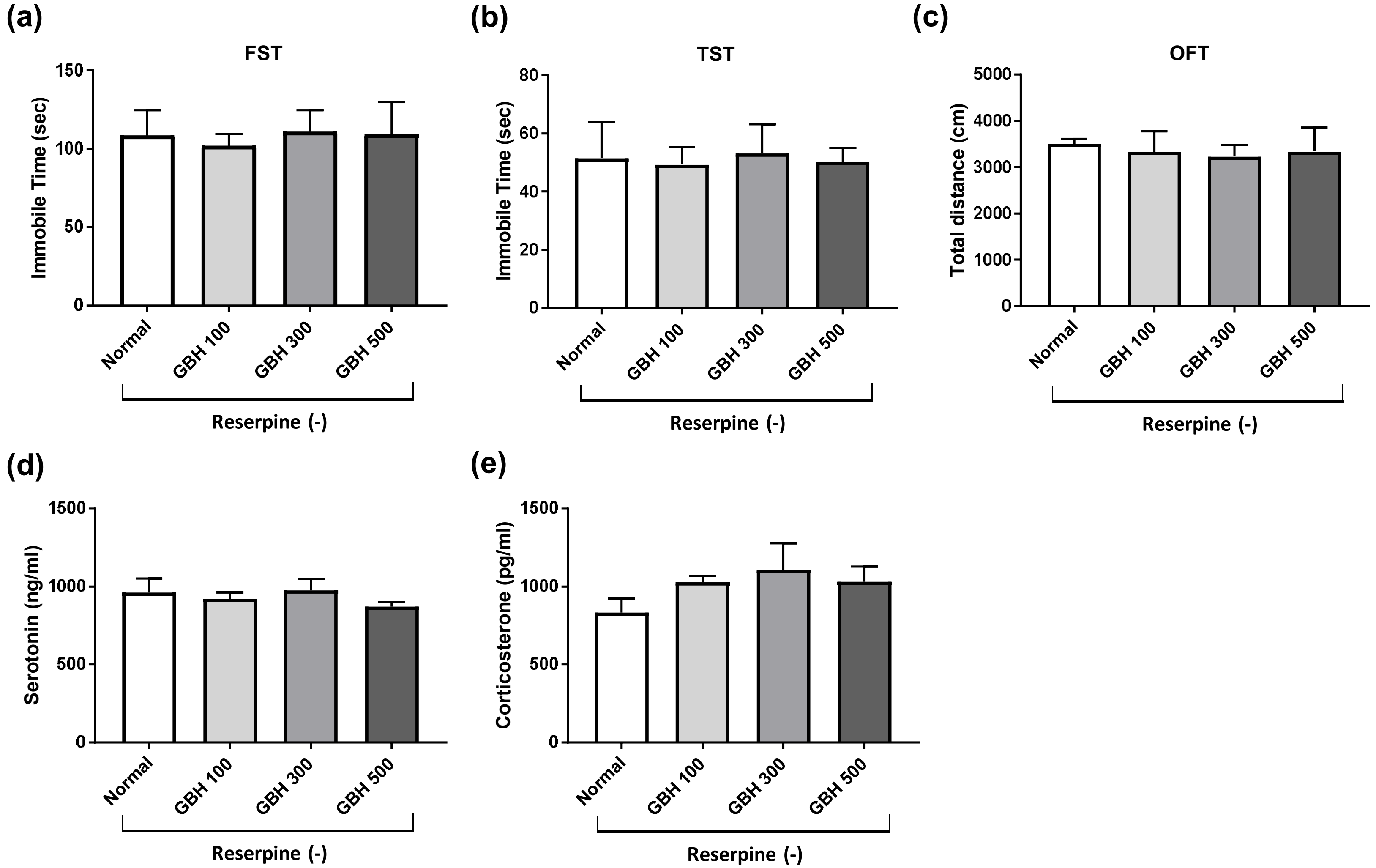

Supplementary Material 1. Effect of on Gyejibokryeong-hwan (GBH) depressive-like behaviors and the concentration of plasma serotonin and corticosterone in mice. (a) Immobility time in the forced swimming test (FST) and (b) tail suspension test (TST); (c) total distance travelled in the open field test (OFT). All behavioral tests were conducted on day 11. The concentration of plasma (d) serotonin and (e) corticosterone was determined by ELISA. Data represent mean ± SD (n = 6, one-way ANOVA).
